# Prognostic risk factors analysis of low-grade gastric intraepithelial neoplasia—a single-center follow-up study

**DOI:** 10.3389/fmed.2025.1637102

**Published:** 2025-08-29

**Authors:** Xin Jiang, Ziting Miao, Xuyu Chen, Jianlei Xia, Bengang Zhou, Yuanyuan Chen, Jingwen Fang, Qiang She, Yaoyao Li, Min Zhang, Yanbing Ding

**Affiliations:** Department of Gastroenterology, The Affiliated Hospital of Yangzhou University, Yangzhou University, Yangzhou, China

**Keywords:** low-grade intraepithelial neoplasia of gastric mucosa, follow-up, regression, risk factors, *H. pylori* infection

## Abstract

**Objective:**

Low-grade gastric intraepithelial neoplasia (LGIN), as a precancerous lesion of gastric cancer, is of great significance in the prevention and treatment of gastric cancer. In this study, we investigated the risk factors associated with LGIN through the follow-up of LGIN patients, and provided a reliable basis for the clinical management of LGIN patients and the formulation of individualized clinical diagnosis and treatment strategies.

**Methods:**

A total of 283 patients, newly diagnosed with LGIN, were enrolled in the study. The regression of LGIN among these patients was assessed by comparing their gastroscopic and pathological findings before and after a rigorous follow-up period. The cohort was then stratified into a progressive group and a non-progressive group. Univariate analysis and multivariate logistic regression analysis were employed to investigate the potential risk factors contributing to the progression of LGIN in these patients.

**Results:**

Among 283 LGIN patients, 8.1% demonstrated lesion progression. Notably, five cases progressed to adenocarcinoma, resulting in an overall cancer incidence rate of 1.8%. Various factors, including age, gender, family history of gastrointestinal tumors, history of alcohol consumption, preference for pickled foods, preference for strong tea, *H. pylori* infection, lesion location, and endoscopic lesion manifestation, were found to be correlated with the progression of LGIN (*p* < 0.05). Multifactorial logistic regression analysis further elucidated that a history of alcohol consumption (*p* = 0.022, OR = 3.224, 95% CI: 1.183–8.782), a family history of gastrointestinal tumors (*p* = 0.029, OR = 3.526, 95% CI: 1.136–10.947), combined with *H. pylori* infection (*p* = 0.024, OR = 4.220, 95% CI: 1.205–14.783), lesion location in the cardia/gastric fundus (*p* = 0.004, OR = 6.838, 95% CI: 1.874–24.958), and endoscopic manifestation of an ulcerated indurated type (*p* = 0.023, OR = 5.073, 95% CI: 1.245–20.667) emerged as significant risk factors for lesion progression in LGIN patients.

**Conclusion:**

LGIN patients with a history of alcohol consumption, a family history of gastrointestinal tumors, a combination of *H. pylori* infection, and lesions located in the cardia/gastric fundus versus lesions endoscopically presenting as ulcerated depressions are more likely to progress to cancer. These risk factors provide a reliable basis for the clinical management of LGIN patients and the development of individualized clinical treatment strategies.

## 1 Introduction

Gastric cancer is a malignant tumor of the digestive tract that arises from a complex interplay of genetic and environmental factors (1). It often has a subtle onset, lacking specific symptoms in its early stages. Consequently, the majority of patients are diagnosed at an advanced stage, with an overall five-year survival rate of less than 50% (2). This poor prognosis poses a grave threat to people’s health and life, highlighting the dire need for effective prevention and control measures against gastric cancer. Therefore, it is imperative at this juncture to implement necessary interventions to decrease the incidence and mortality rates of gastric cancer. In this context, the diagnosis and treatment of gastric precancerous lesions, which are integral to the development process of gastric cancer, have emerged as a focal point of current research.

The developmental progression of gastric cancer is not an overnight occurrence but rather a continuous, multi-stage process involving gradual tissue structural transformations. These stages encompass chronic inflammation, atrophy, intestinal epithelial chemotaxis, heterogeneous hyperplasia, and ultimately carcinoma (3). As the disease advances, the incidence of carcinoma progressively increases at each stage. Notably, heterogeneous hyperplasia can be diagnosed and pathologically graded through pathological biopsies, based on cellular morphology and structural alterations. However, there remains some variability in the diagnosis of heterogeneous hyperplasia associated with carcinoma across different countries and regions. In the 2000 WHO New Classification of Tumors, the term gastric intraepithelial neoplasia (GIN) was introduced to supersede the previous diagnosis of heterogeneous hyperplasia. GIN is categorized into two grades: low-grade gastric intraepithelial neoplasia (LGIN) and high-grade gastric intraepithelial neoplasia (HGIN) (4). Specifically, LGIN corresponds to mild and moderate heterogeneous hyperplasia, whereas HGIN corresponds to severe heterogeneous hyperplasia and carcinoma *in situ*. Patients with HGIN face a higher risk of developing gastric cancer and a greater likelihood of postoperative pathologic escalation compared to those with LGIN. Studies have shown that within a median follow-up period of 4–48 months, 60–85% of HGIN patients progress to gastric cancer, which is approximately 16 times higher than the progression rate for LGIN (5).

Consequently, the current guidelines for managing patients with HGIN are straightforward, emphasizing early surgical or endoscopic treatment. Conversely, the gastric mucosal lesions of patients with LGIN progress slowly, and there is a consensus among scholars that LGIN can be reversed. Research indicates that approximately 38 to 75% of patients’ lesions can regress, with heterogeneity disappearing, while 19 to 50% of patients’ lesions remain stable over an extended period. Only a small proportion of patients may develop progressive gastric cancer (6).

Some current guidelines (7) advocate for early direct endoscopic resection of LGIN to achieve a more precise histopathological diagnosis. Meanwhile, the latest Chinese expert consensus on LGIN management identifies lesions larger than 2 cm and/or those with well-defined borders accompanied by surface microstructural abnormalities as high-risk factors for an upgraded pathological diagnosis. It underscores the importance of re-endoscopy for detailed evaluation and precise biopsy in LGIN patients with these high-risk factors after 3 months. If the lesions remain as LGIN, diagnostic complete resection is recommended (8).

However, undergoing repeated gastroscopies and pathologic biopsies may impose significant physical, psychological, and economic burdens on patients (9). Furthermore, such procedures often lead to a substantial waste of medical resources. Additionally, endoscopic treatment carries high risks of complications such as bleeding and perforation. Consequently, there is currently no unified management approach for low-grade intraepithelial neoplasia (LGIN). Nevertheless, it is worth acknowledging that LGIN, as a precancerous lesion of gastric cancer, holds a certain potential for reversal. By identifying the relevant high-risk factors that influence its progression, appropriate interventions can be implemented. This not only helps to conserve medical resources and alleviate patient suffering but also provides a solid foundation for developing individualized clinical diagnostic and therapeutic strategies tailored to patients’ needs.

The primary endpoints of this study is the prognosis of LGIN patients. The secondary endpoints are the risk factors influencing the progression of LGIN, as well as the correlation between the pathology of LGIN and factors such as age, sex, lesion location, concurrent atrophy and intestinal metaplasia, and *H. pylori*.

## 2 Methods

### 2.1 Study population

The study population comprised 283 patients who presented at the Digestive Endoscopy Center of the Affiliated Hospital of Yangzhou University between June 2017 and June 2022. These patients underwent gastroscopy and were diagnosed with LGIN of the gastric mucosa for the first time based on pathological examination. Inclusion criteria for patients were as follows: (1) individuals aged 18 years or older, with a first-time confirmed diagnosis of LGIN of the gastric mucosa via gastroscopy and pathological biopsy; (2) those who agreed to sign the informed consent form; and (3) patients possessing good communication skills and capable of cooperating with the completion of questionnaires, *H. pylori* testing, and other relevant examinations. Exclusion criteria included: (1) individuals with severe diseases of vital organs such as the heart, lungs, brain, or kidneys, or those with psychiatric disorders; (2) patients with a previous pathological diagnosis of HGIN, gastric cancer, or a history of other metastatic gastric tumors; and (3) those who had undergone previous gastric surgery.

### 2.2 Collection of general clinical data

Our epidemiological questionnaire mainly includes the following aspects: Basic information (name, gender, age, place of residence, contact details, height, weight), smoking history, drinking history, tea consumption history, intake of fresh vegetables and fruits, intake of pickled foods and other dietary and living habits, family history of digestive tract tumors, history of gastric diseases, previous disease history, previous gastroscopy and *H. pylori* infection status, etc. Among them, the smoking history is defined as: an average daily smoking volume of ≥1 cigarette, with a duration of more than 6 months; A drinking history is defined as an alcohol consumption of ≥20 g/d or a continuous drinking period of more than 2 years. The history of drinking strong tea is defined as consuming more than 3 cups of very strong tea brewed every day for a period of more than 3 months. Record whether the included population likes hot food, whether the eating speed is too fast (each meal takes less than 15 min), and for other eating habits such as the intake of fresh vegetables and fruits, pickled products, spicy and stimulating foods, and smoked, fried and grilled foods, if the intake is more than 3 times a week, it is defined as liking; otherwise, it is defined as disliking.

### 2.3 Gastroscopy and histopathological examination

Endoscopists conducted endoscopic examinations on all study subjects in accordance with the standard operating procedures for gastroscopy, meticulously documenting the locations of gastric mucosal lesions, which were categorized into cardia/fundus, body, angle, and antrum of the stomach. According to the different endoscopic manifestations of the lesion, it was further classified into four groups: the hyperplastic and raised type, the rough and erosive type, the ulcerative and depressed type, and the flat and superficial type. Among them, the hyperplastic and raised type refers to the lesion presenting as polyp and mass protrusions, with a height greater than 0.5 cm. The rough erosive type refers to the endoscopic manifestation of rough mucosal protrusion, but with a height of less than 0.5 cm, accompanied by congestion, erosion and granular changes. Ulcerative depressed type refers to the lesion presenting ulcerative changes, being depressed inward and having a depth greater than 0.5 cm. The flat superficial type refers to the situation where only the mucosal surface shows redness or mottled changes under endoscopy, without the above-mentioned related changes. The pathological results were strictly evaluated in accordance with the relevant pathological diagnostic criteria of the Chinese consensus on Chronic Gastritis and the Intuitive Analogue scale of the New Sydney System for five histological changes. The degree of histological change assessment was classified into four grades: none, mild, moderate and severe. Suspicious lesions underwent tissue biopsy and were subsequently sent to the pathology department for processing and diagnosis. Two senior pathologists independently reviewed and diagnosed the pathological sections. These patients were followed up, with the initial detection of low-grade intraepithelial neoplasia in the gastric mucosa serving as the baseline status. Subsequent follow-up examinations involved biopsy sampling from the corresponding sites. For any newly identified lesions during follow-up, rigorous endoscopic examinations and pathological biopsies were conducted in accordance with the aforementioned protocols.

### 2.4 Detection of *H. pylori* infection

All enrolled subjects underwent rapid urease testing and a ^13^C-urea breath test. A positive result in either test was considered indicative of *H. pylori* infection. If the rapid urease test result was negative, patients were instructed to discontinue any medications that could affect the breath test, such as proton pump inhibitors and antibiotics, for 1 month before undergoing a follow-up ^13^C-urea breath test. The final diagnostic criterion was based on the ^13^C-urea breath test results, where a DOB value greater than 4.0 was considered indicative of *H. pylori* infection, and any value below this threshold was deemed as negative for *H. pylori* infection.

### 2.5 Follow-up of patient cases

We conducted rigorous follow-up on the enrolled subjects through outpatient visits, telephone calls, and WeChat. Endoscopic examinations were repeated at intervals of approximately 3–6 months or 1 year, with the final gastroscopy follow-up serving as the endpoint for assessment of disease outcome. The criteria for assessing the outcome of LGIN are as follows: (1) Lesion reversal: the lesion is reduced or disappears compared to the previous state; (2) Lesion stability: no change in the lesion grade; (3) Lesion progression: an increase in lesion grade compared to the previous state, upgrading to HGIN, or even the occurrence of malignant transformation.

### 2.6 Statistical analyses

We utilized SPSS version 25.0 software to conduct comprehensive statistical analysis on all collected data. For data conforming to normal distribution, the T-test method was employed for analysis, with results presented as (mean ± standard deviation). Chi-square or rank-sum tests were performed on categorical and ordinal data. Spearman’s correlation analysis was utilized for assessing the correlation between relevant factors. Comparisons of rates among related data were conducted using chi-square tests. We included the risk factors with a *p*-value less than 0.05 in the univariate chi-square analysis into the multivariate logistic regression analysis. Logistic regression analysis was applied to investigate risk factor variables. A *p*-value less than 0.05 was considered statistically significant.

## 3 Results

### 3.1 General characteristics

#### 3.1.1 Gender and age distribution of LGIN patients

A total of 283 LGIN patients were included in this study, including 206 males and 77 females, the oldest of whom was 85 years old and the youngest was 29 years old, with a mean age of 57.12 ± 10.19 years. The follow-up time of their gastroscopy was more than 3 months, and the follow-up period was good, no patients were lost to follow-up with an average follow-up time of 19.56 ± 15.01 months (Table 1).

**Table 1:** Gender and age distribution of 283 LGIN patients.

Characteristics	*n*	Proportion (%)	Mean age
Gender			
Male	206	72.8	57.27 ± 9.90
Female	77	27.2	56.71 ± 11.01
Age			
<45	30	10.6	38.17 ± 4.86
45–59	146	51.6	53.82 ± 4.20
60–74	98	34.6	65.88 ± 4.51
≥75	9	3.2	78.44 ± 3.58
	283	100	57.12 ± 10.19

#### 3.1.2 The distribution of lesion sites in LGIN patients

Among the 283 patients, 34 (12.0%) were located in the cardia/ gastric fundus. 19 cases were located in the gastric body, accounting for 6.7%; 88 cases were located in the stomach angle, accounting for 31.1%; There were 142 cases located in the gastric antrum, accounting for 50.2%, so the gastric mucosal lesions in LGIN patients were mainly in the gastric antrum, followed by the stomach angle, and the cardia/ gastric fundus and the gastric body were relatively rare. We found that there were no significant statistical differences in the distribution of lesions, age and sex of different lesions, and there were no significant differences in the distribution of lesions, age and sex (*p* > 0.05) (Tables 2, 3).

**Table 2:** The distribution of lesion sites in LGIN patients of the corresponding age groups.

Location	Age groups					*p*
	<45 (*n* = 30)	45–59 (*n* = 146)	60–75 (*n* = 98)	≥75 (*n* = 9)	Total (*n* = 283)	
Cardia/gastric fundus	3 (10.0%)	18 (12.3%)	10 (10.2%)	3 (33.3%)	34 (12.0%)	0.650
Gastric body	1 (3.3%)	10 (6.9%)	8 (8.2%)	0 (0%)	19 (6.7%)	
Gastric angle	11 (36.7%)	44 (30.1%)	32 (32.6%)	1 (11.1%)	88 (31.1%)	
Gastric antrum	15 (50.0%)	74 (50.7%)	48 (49.0%)	5 (55.6%)	142 (50.2%)	

**Table 3:** The distribution of lesion sites in LGIN patients of the corresponding sex groups.

Location	sex groups			*p*
	Male (*n* = 206)	Female (*n* = 77)	Total (*n* = 283)	
Cardia/gastric fundus	26 (12.6%)	8 (10.4%)	34 (12.0%)	0.842
Gastric body	13 (6.3%)	6 (7.8%)	19 (6.7%)	
Gastric angle	66 (32.1%)	22 (28.6%)	88 (31.1%)	
Gastric antrum	101 (49.0%)	41 (53.2%)	142 (50.2%)	

#### 3.1.3 Pathological grade composition of LGIN patients

Among the 283 LGIN patients, 243 patients with mild dysplasia and 40 patients with moderate dysplasia were in the pathological grade, with a ratio of 1:6.1, among which the patients with moderate dysplasia were mainly middle-aged and elderly people, and their proportion increased by age. Our analysis found that there were statistically significant differences between different age groups and pathological grades (*p* < 0.05) (Table 4). In addition, we further explored the correlation between different pathological grades and the occurrence sites of each lesion, and found that the lesions of patients with mild dysplasia were mainly located in the antrum and gastric angle, while the lesions of patients with moderate dysplasia were more common in the gastric body and cardia/fundus, and there was a certain statistical difference between different lesion sites and LGIN pathological grades (*p* < 0.05) (Table 5).

**Table 4:** The composition of pathological grades in different age groups of LGIN patients.

Pathological grade	Age groups					*p*
	<45 (*n* = 30)	45–59 (*n* = 146)	60–74 (*n* = 98)	≥75 (*n* = 9)	Total (*n* = 283)	
Mild dysplasia	28 (93.3%)	129 (88.4%)	81 (82.7%)	5 (55.6%)	243 (85.9%)	0.021
Moderate dysplasia	2 (6.7%)	17 (11.6%)	17 (17.3%)	4 (44.4%)	40 (14.1%)	

**Table 5:** The composition of pathological grades of different lesion sites in LGIN patients.

Pathological grade	Lesion site groups				*p*
	Cardia/gastric fundus (*n* = 34)	Gastric body (*n* = 19)	gastric angle (*n* = 88)	gastric antrum (*n* = 142)	
Mild dysplasia	26 (76.5)	13 (68.4)	80 (90.9)	124 (87.3)	0.025
Moderate dysplasia	8 (23.5)	6 (31.6)	8 (9.1)	18 (12.7)	

#### 3.1.4 Histopathology of gastric mucosa with atrophy and intestinal metaplasia in LGIN patients

Among the 283 patients with LGIN, 57.2% (162/283) had atrophy, 42.8% (121/283) had no atrophy, 77.0% (218/283) had intestinal metaplasia, and 23.0% (65/283) had no intestinal metaplasia. We found that atrophy and intestinal metaplasia were mainly mild and moderate, and the incidence of atrophy and intestinal metaplasia gradually increased with age (*p* < 0.05), but there was no significant difference in the grade of atrophy and intestinal metaplasia at different ages (*p* > 0.05) (Table 6).

**Table 6:** Histopathology of gastric mucosa with atrophy and intestinal metaplasia in LGIN patients.

Characteristics	Age groups					*p*
	<45 (*n* = 30)	45-59 (*n* = 146)	60-74 (*n* = 98)	≥75 (*n* = 9)	Total (*n* = 283)	
Atrophy						
No	15 (50.0%)	71 (48.6%)	34 (34.7%)	1 (11.1%)	121 (42.8%)	0.029
Yes	15 (50.0%)	75 (51.4%)	64 (65.3%)	8 (88.9%)	162 (57.2%)	
Atrophy grade						
None	15 (50.0%)	71 (48.6%)	34 (34.7%)	1 (11.1)	121 (42.8%)	0.090
Mild	13 (43.3%)	44 (30.2%)	43 (43.9%)	6 (66.7%)	106 (37.4%)	
Moderate	2 (6.7%)	25 (17.1%)	17 (17.3%)	1 (11.1%)	45 (15.9%)	
Severe	0 (0%)	6 (4.1%)	4 (4.1%)	1 (11.1%)	11 (3.9%)	
Intestinal metaplasia						
No	13 (43.3%)	33 (22.6%)	18 (18.4%)	1 (11.1%)	65 (23.0%)	0.030
Yes	17 (56.7%)	113 (77.4%)	80 (81.6%)	8 (88.9%)	218 (77.0%)	
Intestinal metaplasia grade						
None	13 (43.3%)	33 (22.6%)	18 (18.3%)	1 (11.1%)	65 (23.0%)	0.269
Mild	8 (26.7%)	63 (43.1%)	42 (42.9%)	4 (44.5%)	117 (41.3%)	
Moderate	6 (20.0%)	42 (28.8%)	29 (29.6%)	3 (33.3%)	80 (28.3%)	
Severe	3 (10.0%)	8 (5.5%)	9 (9.2%)	1 (11.1%)	21 (7.4%)	

#### 3.1.5 *H. pylori* infection among different groups of LGIN patients

In this study, all 283 LGIN patients completed at least one ^13^C breath test, and a total of 146 *H. pylori* positive infections were detected, with a positive rate of 51.6% (146/283). We found no statistically significant differences between age groups and *H. pylori* infection (*p* > 0.05). Among the different lesions, *H. pylori* infection was more common in the gastric antrum, followed by the gastric angle and cardia/gastric fundus, while the positive rate of *H. pylori* infection in the gastric body was relatively low, but there was no significant statistical difference between lesion locations and *H. pylori* infection (*p* > 0.05). In addition, we found that *H. pylori* infection was significantly correlated with gastric mucosal atrophy and atrophy grade (*p* < 0.01), and was significantly associated with the gastric mucosal intestinal metaplasia and intestinal metaplasia grade (*p* < 0.05) (Table 7).

**Table 7:** *H. pylori* infection among different groups of LGIN patients.

Characteristics	*H. pylori* positive (*n*1 = 146)	*H. pylori* negative (*n*2 = 137)	Total (*n* = 283)	*p*
Age				
<45	16 (53.3%)	14 (46.7%)	30	0.673
45–59	71 (48.6%)	75 (51.4%)	146	
60–74	55 (56.1%)	43 (43.9%)	98	
≥75	4 (55.6%)	5 (44.4%)	9	
Lesion location				
Cardia/gastric fundus	16 (47.1%)	18 (52.9%)	34	0.324
Gastric body	7 (36.8%)	12 (63.2%)	19	
Gastric angle	43 (48.9%)	45 (51.1%)	88	
Gastric antrum	80 (56.3%)	62 (43.7%)	142	
Atrophy				
No	43 (35.5%)	78 (64.5%)	121	0.001
Yes	103 (63.6%)	59 (36.4%)	162	
Atrophy grade				
None	43 (35.5%)	78 (64.5%)	121	0.001
Mild	61 (57.5%)	45 (42.5%)	106	
Moderate	36 (80.0%)	9 (20.0%)	45	
Severe	6 (54.5%)	5 (45.5%)	11	
Intestinal metaplasia				
No	24 (36.9%)	41 (63.1%)	65	0.007
Yes	122 (56.0%)	96 (44.0%)	218	
Intestinal metaplasia grade				
None	24 (36.9%)	41 (63.1%)	65	0.016
Mild	60 (51.3%)	57 (48.7%)	177	
Moderate	51 (63.7%)	29 (36.3%)	80	
Severe	11 (52.4%)	10 (47.6%)	21	

### 3.2 Prognosis of LGIN patients at final follow-up

In this study, 283 LGIN patients completed 2 or more gastroscopies and pathological biopsies, with an average follow-up time of 19 months, and finally 171 LGIN patients had reversed gastric mucosal lesions, accounting for 60.4% (171/283), 89 cases of gastric mucosal lesions remained stable, accounting for 31.5% (89/283), 23 cases of gastric mucosal lesions progressed, accounting for 8.1% (23/283), of which 5 cases progressed to adenocarcinoma, and the total carcinogenesis rate was 1.8% (5/283). The reversal rate and carcinogenesis rate were 62.6% (152/243) and 0.4% (1/243) in the mild dysplasia group, and the reversal rate and carcinogenesis rate were 47.5% (19/40) and 10.0% (4/40) in the moderate dysplasia group (Table 8).

**Table 8:** The final outcome of LGIN patients with different pathological grades.

Pathological grade	*n*	Reversion	Mild dysplasia	Moderate dysplasia	HGIN	Cancer
Mild dysplasia	243	152 (62.6%)	71 (29.2%)	10 (4.1%)	9 (3.7%)	1 (0.4%)
Moderate dysplasia	40	19 (47.5%)	0 (0%)	8 (20.0%)	9 (22.5%)	4 (10.0%)

### 3.3 Univariate analysis of the progression of gastric mucosal lesions in LGIN patients

We defined patients with reversed or stable gastric mucosal lesions as the non-progression group and patients with pathological escalation as the progression group, and found that the progression of LGIN was significantly related to age, gender, drinking history, preference for pickled products, preference for strong tea, family history of gastrointestinal tumors, lesion location, endoscopic manifestations, and *H. pylori* infection (*p* < 0.05) (Table 9).

**Table 9:** Univariate analysis of the progression of gastric mucosal lesions in LGIN patients.

Characteristics	Non-progression group (*n* = 260)	Progression group (*n* = 23)	Total (*n* = 283)	*p*
Age				0.011
<45	30 (11.5%)	0 (0%)	30 (10.6%)	
45–59	136 (52.3%)	10 (43.5%)	146 (51.6%)	
60–74	88 (33.9%)	10 (43.5%)	98 (34.6%)	
≥75	6 (2.3%)	3 (13.0%)	9 (3.2%)	
Sex				0.037
Male	185 (71.2%)	21 (91.3%)	206 (72.8%)	
Female	75 (28.8%)	2 (8.7%)	77 (27.2%)	
Residence				0.256
Urban	145 (55.8%)	10 (43.5%)	155 (54.8%)	
Rural	115 (44.2%)	13 (56.5%)	128 (45.2%)	
Smoking history				0.070
No	163 (62.7%)	10 (43.5%)	173 (61.1%)	
Yes	97 (37.3%)	13 (56.5%)	110 (38.9%)	
Drinking history				0.016
No	196 (75.4%)	12 (52.2%)	208 (73.5%)	
Yes	64 (24.6%)	11 (47.8%)	75 (26.5%)	
Dietary habits				
Irregular diet	16 (6.2%)	2 (8.7%)	18 (6.4%)	0.648
Regular diet	244 (93.8%)	21 (91.3%)	265 (93.6%)	
Food temperature				
Too hot	25 (9.6%)	3 (13.0%)	28 (9.9%)	0.485
Not too hot	235 (90.4%)	20 (87.0%)	255 (90.1%)	
Feeding rate				
Too fast	51 (19.6%)	5 (21.7%)	56 (19.8%)	0.787
Not too fast	209 (80.4%)	18 (78.3%)	227 (80.2%)	
Spicy and irritating food				
Yes	48 (18.5%)	2 (8.7%)	50 (17.7%)	0.390
No	212 (81.5%)	21 (91.3%)	233 (82.3%)	
Deep-fried barbecue food				
Yes	23 (8.8%)	1 (4.3%)	24 (8.5%)	0.705
No	237 (91.2%)	22 (95.7%)	259 (91.5%)	
Pickled food				
Yes	79 (30.4%)	12 (52.2%)	91 (32.2%)	0.032
No	181 (69.6%)	11 (47.8%)	192 (67.8%)	
Strong tea				
Yes	37 (14.2%)	7 (30.4%)	44 (15.5%)	0.040
No	223 (85.8%)	16 (69.6%)	239 (84.5%)	
Inadequate intake of fresh fruits and vegetables				
Yes	68 (26.2%)	4 (17.4%)	72 (25.4%)	0.355
No	192 (73.8%)	19 (82.6%)	211 (74.6%)	
Family history of gastrointestinal cancer				0.011
Yes	34 (13.1%)	8 (34.8%)	42 (14.8%)	
No	226 (86.9%)	15 (65.2%)	241 (85.2%)	
Gastric polyps				
Yes	64 (24.6%)	5 (21.7%)	69 (24.4%)	
No	196 (75.4%)	18 (78.3%)	214 (75.6%)	0.758
Gastric ulcer				
Yes	21 (8.1%)	3 (13.0%)	24 (8.5%)	
No	239 (91.9%)	20 (87.0%)	259 (91.5%)	0.427
Lesion location				0.004
Cardia / gastric fundus	26 (10.0%)	8 (34.8%)	34 (12.0%)	
Gastric body	17 (6.5%)	2 (8.7%)	19 (6.7%)	
Gastric angle	82 (31.5%)	6 (26.1%)	88 (31.1%)	
Gastric antrum	135 (52.0%)	7 (30.4%)	142 (50.2%)	
Endoscopic morphology				0.001
Hypertrophic bulges	72 (27.7%)	4 (17.4%)	76 (26.9%)	
Rough erosion	139 (53.5%)	8 (34.8%)	147 (51.9%)	
Ulcer dimpling	24 (9.2%)	10 (43.5%)	34 (12.0%)	
Flat and shallow	25 (9.6%)	1 (4.3%)	26 (9.2%)	
Atrophy				0.714
Yes	148 (56.9%)	14 (60.9%)	162 (57.2%)	
No	112 (43.1%)	9 (39.1%)	121 (42.8%)	
Intestinal metaplasia				0.055
Yes	204 (78.5%)	14 (60.9%)	218 (77.0%)	
No	56 (21.5%)	9 (39.1%)	65 (23.0%)	
*H. pylori* infection				0.002
Positive	127 (48.8%)	19 (82.6%)	146 (51.6%)	
Negative	133 (51.2%)	4 (17.4%)	137 (48.4%)	

### 3.4 Multivariate logistic regression analysis of the progression of gastric mucosal lesions in LGIN patients

We included nine variables selected from the univariate analysis results, namely age, gender, drinking history, preference for pickled foods/strong tea, family history of gastrointestinal tumors, lesion location, endoscopic morphology, and *H. pylori* infection, in the multivariate logistic regression analysis. The results showed that drinking history (*p* = 0.022, OR = 3.224, 95% CI: 1.183–8.782), family history of gastrointestinal tumors (*p* = 0.029, OR = 3.526, 95% CI: 1.136–10.947), *H. pylori* infection (*p* = 0.024, OR = 4.220, 95% CI: 1.205–14.783), lesion located in the cardia/gastric fundus (*p* = 0.004, OR = 6.838, 95% CI: 1.874–24.958), and endoscopic appearance of Ulcer dimpling type (*p* = 0.023, OR = 5.073, 95% CI: 1.245–20.667) were risk factors for the progression of LGIN (Table 10).

**Table 10:** Multivariate logistic regression analysis of the progression of gastric mucosal lesions in LGIN patients.

Characteristics	Risk factors	B	Sig (*p*)	OR	95%CI
Drinking history	Yes vs. No	1.171	0.022	3.224	1.183–8.782
Family history of gastrointestinal cancer	Yes vs. No	1.260	0.029	3.526	1.136–10.947
*H. pylori* infection	Yes vs. No	1.440	0.024	4.220	1.205–14.783
Lesion location	Cardia/gastric fundus	1.923	0.004	6.838	1.874–24.958
	Gastric angle	0.393	0.679	1.482	0.229–9.572
	Gastric body	0.134	0.831	1.143	0.333–3.924
	Gastric antrum (Reference)	-	-	1	-
Endoscopic morphology					
	Ulcer dimpling	1.624	0.023	5.073	1.245–20.667
	Rough erosion	0.206	0.762	0.814	0.215–3.080
	Flat and shallow	0.170	0.885	0.844	0.084–8.479
	Hypertrophic bulges (Reference)	-	-	1	-

No Drinking history, no family history of gastrointestinal tumors, no *H. pylori* infection, lesions located in the gastric antrum, and hypertrophic bulges type were used as reference value.

## 4 Discussion

Gastric cancer is a highly lethal malignant tumor of the digestive system and represents a significant and persistent threat to people’s health (10). Therefore, early detection, diagnosis, and treatment are paramount in its prevention and control (11). LGIN, as a crucial component in Correa’s cascade for gastric cancer, is also key to its prevention and treatment. Similar to HGIN, LGIN is a well-defined non-invasive neoplastic lesion, primarily characterized by morphological changes in tissue structure and cellular atypia. In the past, terms such as “dysplasia” and “atypical hyperplasia” were used to describe it, but dysplasia mainly emphasizes the changes in cellular and tissue structural atypia, while intraepithelial neoplasia focuses more on the process of tumorigenesis (12). Therefore, intraepithelial neoplasia is more suitable for describing precancerous lesions. Although LGIN has a degree of reversibility, there is also a significant possibility of missed diagnosis and pathological upgrading. Hence, it should be highly prioritized in clinical practice. Comprehensive analysis should be conducted based on patients’ clinical symptoms, endoscopic manifestations, and pathological biopsy results during follow-up to develop individualized treatment and follow-up plans, rather than blindly performing endoscopic examinations and repeated pathological biopsies. Our study aims to comprehensively analyze the clinical, pathological, and epidemiological data of patients with LGIN diagnosed at our hospital and evaluate their clinical outcomes and related influencing factors.

In this study, we found that LGIN mainly affects middle-aged and elderly individuals. The latest epidemiological analysis of gastric cancer (13) indicates that the incidence of gastric cancer remains low before age 40, then rapidly increases thereafter, peaking in both men and women over 80 years old. Since LGIN precedes gastric cancer as a precancerous stage, the age of onset in our study aligns well with the age characteristics of gastric cancer incidence. In terms of gender, the male-to-female ratio in our study was 2.68:1, and gender differences were statistically significant in univariate analysis of LGIN progression. Considering that males are often at higher risk of exposure to smoking, alcohol consumption, and poor lifestyle habits, and are influenced by various genetic factors, differences in sex hormone levels, dietary habits, etc., these may contribute to the gender disparity in LGIN patient distribution (14). Among the factors contributing to the development of gastric cancer, poor lifestyle and dietary habits are also among the most significant risks. A large-sample Meta-analysis conducted abroad (15) studied 23 projects on smoking and gastric cancer risk from North America, Europe, and Asia, finding that current and former smokers had a 1.25-fold and 1.20-fold increased risk of gastric cancer, respectively, compared to nonsmokers. Additionally, the risk of gastric cancer gradually increases with the number of cigarettes smoked per day and the duration of smoking. Similarly, alcohol stimulation can lead to erosive damage to the gastric mucosa, potentially inducing ulcers and even cancer. The latest Meta-analysis on the association between alcohol consumption and gastric cancer risk in the general population (16) revealed that individuals with a history of alcohol consumption have a 1.20-fold increased risk of gastric cancer compared to nondrinkers, demonstrating a clear dose–response relationship. In our study, we found that alcohol consumption is a risk factor for LGIN progression. Therefore, for male patients, abstaining from alcohol is one of the effective means to prevent the progression of LGIN.

In terms of dietary habits, we observed that irregular eating patterns, consumption of excessively hot foods, preference for smoked and fried foods, fondness for spicy and pungent foods, and inadequate intake of fresh vegetables and fruits all constitute a certain proportion among patients with LGIN; however, none of these factors were significantly associated with the progression of LGIN. Conversely, consuming pickled products more than three times per week and a preference for drinking more than three cups of strong tea per day were found to be correlated with the progression of LGIN. Ren et al. (17) conducted a meta-analysis study, which revealed that a diet high in pickled products significantly increases the risk of gastric cancer in East Asia, particularly prominent in China and South Korea. Nonetheless, studies on the association between tea consumption and gastric cancer progression are relatively scarce and conflicting. Generally, tea is rich in active substances such as tea polyphenols and alkaloids, which are believed to scavenge free radicals, alleviate fatigue, exhibit anti-inflammatory and antioxidant effects, thereby providing protective benefits to the body. However, some studies have indicated that tea consumption does not reduce the risk of cancer, and drinking more than 4 grams of strong tea per day significantly increases the risk of gastric cancer (18). This may be related to the stimulating effect of caffeine and other substances in tea on excessive gastric acid secretion. The local residents in the study area have a penchant for pickled vegetables, preserved vegetables, salted meat, and strong tea. Therefore, the findings of this study possess certain regional representativeness.

In terms of genetic factors, a family history of cancer has long been recognized as a significant risk factor for gastric cancer. In the study region, which is an area with a high incidence of upper gastrointestinal cancer, individuals with a family history of digestive tract tumors comprised 14.8% of the study population. Multivariate analysis results further indicate that a family history of digestive tract tumors is an important risk factor for the progression of LGIN. This association may be mediated by genetic polymorphisms regulating the homeostasis pathways of the gastric mucosa, such as pro-inflammatory cytokines (19), tumor suppressor genes (20), and genes involved in acid secretion (21). Clinically, patients with a family history usually present with an early onset, accelerated development into atypical hyperplasia (22). Therefore, patients with LGIN who have a family history of digestive tract tumors should be closely monitored and undergo regular follow-up evaluations.

*H. pylori*, recognized by the World Health Organization (WHO) as a Group I carcinogen, is a significant contributor to gastric cancer. In recent years, with the increasing drug resistance rates, the eradication rate of *H. pylori* infection has gradually declined (23). In the present study, 51.6% (146/283) of patients with LGIN had concurrent *H. pylori* infection, indicating that the *H. pylori* infection rate among residents in our region remains at a relatively high level. Multivariate regression analysis further confirmed that *H. pylori* infection is a high-risk factor for the progression of LGIN lesions. Therefore, it is crucial to improve *H. pylori* detection in gastric cancer screening. However, studies (24) have shown that while *H. pylori* eradication can reverse gastric mucosal atrophy, it fails to halt the progression of intestinal metaplasia and LGIN. Although there is ongoing controversy regarding whether *H. pylori* eradication can reverse intraepithelial neoplasia, both domestic and international guidelines recommend eradication therapy for those with concurrent *H. pylori* infection. Therefore, all patients with positive *H. pylori* infection successfully underwent quadruple therapy for eradication treatment subsequently. Consequently, clinical practice should emphasize the importance of complete *H. pylori* testing, assessment of eradication indications, and, when necessary, the implementation of resistance gene testing to develop individualized eradication treatment plans (25–31).

This study analyzed the endoscopic data of patients with LGIN and found that the lesions were most frequently distributed in the antropyloric region, followed by the angularis pyloricus, while the cardia/fundus and corpus regions were relatively less affected. This distribution aligns well with the preferred sites of gastric cancer development. The research further revealed an association between different pathological grades and lesion locations, with mild dysplasia predominantly located in the antropyloric region and angularis pylorus, while moderate dysplasia accounted for a larger proportion in the cardia/corpus regions. Moreover, this study discovered a significant correlation between lesion location and LGIN progression. Specifically, patients with LGIN lesions in the cardia had a 6.838-fold increased risk of ultimate lesion progression compared to those with lesions in the antropyloric region. Therefore, special attention should be paid to lesions in the proximal stomach, especially the cardia, during gastroscopic examinations to avoid missed diagnoses. Beyond lesion location, this study also analyzed the endoscopic manifestations of the patients. Studies have shown that whitish appearance, irregular margins, marked IM, and histological diagnosis of LGIN more than twice within the first year were predictors for progression (32). The lesions primarily presented as rough and erosive types endoscopically, followed by hyperplastic and elevated types, while ulcerated and depressed types, as well as flat and superficial types, were relatively less common. Additionally, this study found that LGIN progression was associated with its endoscopic manifestations. Lesions with ulcerated and depressed types were more likely to progress compared to those with hyperplastic and elevated types. Although both elevated and ulcerative lesions are relatively easy to detect endoscopically, endoscopists should pay close attention during the examination process. For ulcerated and depressed lesions, careful multi-point biopsy sampling can improve lesion detection rates and prevent missed diagnoses that may lead to disease progression.

Precancerous states, akin to precancerous lesions, represent a crucial aspect in the prevention and control of gastric cancer. Intestinal metaplasia often accompanies atrophic gastritis, and this study analyzed the pathological characteristics of LGIN. It was found that 57.2% (162/283) of LGIN cases were accompanied by atrophy, and 77.0% (218/283) were accompanied by intestinal metaplasia. The atrophy and intestinal metaplasia were predominantly mild to moderate in severity and positively correlated with age, with an increasing proportion observed as age advanced. Some studies posit that intestinal metaplasia is the sole prerequisite for the development of intestinal-type gastric cancer (33). Intestinal metaplasia is primarily classified into two subtypes: complete and incomplete. The complete type, also known as type I or small intestinal type, consists mainly of mature absorptive cells and goblet cells, whereas the incomplete type comprises columnar intermediate cells, immature goblet cells, and a very small number of absorptive cells. Based on the difference in mucin secreted by intermediate cells, the incomplete type is further subclassified into type II, which secretes a mixture of salivary and neutral mucins, and type III, which secretes sulfomucin. It is believed that the incomplete type of intestinal metaplasia carries a higher risk of gastric cancer development (34, 35). A recent 20-year follow-up study (36) of patients with precancerous lesions of the stomach at high risk for gastric adenocarcinoma in a Hispanic population indicated that *H. pylori* eradication therapy can halt the histological progression to gastric adenocarcinoma. Moreover, compared to complete intestinal metaplasia, the risk of progression to gastric cancer is up to 13.4 times higher in incomplete intestinal metaplasia, suggesting that incomplete intestinal metaplasia is a potent predictor of gastric cancer. In our study, a significant correlation was observed between *H. pylori* and the occurrence of atrophy and intestinal metaplasia, and *H. pylori* infection was also closely related to the grade of atrophy and intestinal metaplasia. However, no statistical difference was found between LGIN progression and the presence of concurrent atrophy and intestinal metaplasia (*p* > 0.05). This discrepancy with previous studies may be attributed to the small sample size in our study, leading to potential bias in the results, or it may be related to the higher proportion of complete intestinal metaplasia observed. Nevertheless, it cannot be denied that atrophy and intestinal metaplasia serve as the foundation for the occurrence of LGIN. Therefore, for LGIN accompanied by atrophy and intestinal metaplasia, further subclassification should be made pathologically, distinguishing between metaplastic atrophy and non-metaplastic atrophy, as well as between complete and incomplete intestinal metaplasia. When necessary, specific chemical staining techniques and related molecular markers should be utilized for diagnosis, to facilitate the management and follow-up of LGIN patients and the assessment of the risk of gastric cancer progression.

Although there are limited studies on the follow-up outcomes of LGIN, it is generally acknowledged that the risk of progression increases exponentially with the severity of dysplasia. In this study, 283 LGIN patients were followed up for an average of 19 months. Ultimately, 60.4% (171/283) of patients experienced lesion reversal, 31.5% (89/283) had stable lesions, and 8.1% (23/283) exhibited lesion progression. The overall canceration rate was 1.8% (5/283). Among patients, those with mild dysplasia had a reversal rate of 62.6% (152/243) and a canceration rate of 0.4% (1/243), whereas those with moderate dysplasia had a reversal rate of 47.5% (19/40) and a canceration rate of 10.0% (4/40). These results indicate that although LGIN has the potential for reversal, there is a non-negligible risk of canceration. Furthermore, the more risk factors associated with LGIN, the greater the possibility of progression. Therefore, close follow-up is of utmost importance.

The significance of this study is that we conducted long-term close clinical follow-up of LGIN patients, explored the risk factors affecting the prognosis of LGIN patients. By comprehensively analyzing the clinical, endoscopic, and pathological data of LGIN patients treated in our hospital, we found that age, gender, poor dietary habits such as alcohol consumption, consumption of strong tea, preference for pickled foods, family history of digestive tract cancer, lesion location, endoscopic manifestations, and *H. pylori* infection were significantly correlated with the prognosis of LGIN. Multivariate analysis further confirmed that alcohol consumption, family history of digestive tract cancer, lesion location, and endoscopic manifestations were risk factors for LGIN progression. Therefore, in clinical practice, for LGIN patients, we should focus on these risk factors, develop individualized treatment and follow-up plans, reduce the risk of progression to gastric cancer, and improve patient prognosis.

However, our study had certain limitations. As a retrospective single-center clinical study, our study had relatively small sample sizes, which may lead to statistical limitations caused by potential overfitting and the occurrence of a few events. Moreover, the epidemiological investigation data have certain regional limitations because the patients included in our study were from East Asia and have not been further finely quantified and stratified regarding smoking, drinking, and related lifestyle and dietary habits. It is impossible to determine whether there is a correlation between LGIN and its dosage frequency, nor has a follow-up study on the eradication of *H. pylori* been conducted again. Subsequently, we will continue to expand the sample size, extend the follow-up period, and conduct multi-center prospective studies for further verification.

## Data Availability

The raw data supporting the conclusions of this article will be made available by the authors, without undue reservation.
